# Forecasting Tourist Arrivals for Hainan Island in China with Decomposed Broad Learning before the COVID-19 Pandemic

**DOI:** 10.3390/e25020338

**Published:** 2023-02-12

**Authors:** Jingyao Chen, Jie Yang, Shigao Huang, Xin Li, Gang Liu

**Affiliations:** 1 School of Business, Macau University of Science and Technology, Macau SAR, China; 2 College of Artificial Intelligence, Chongqing Industry & Trade Polytechnic, Chongqing 408000, China; 3 Faculty of Health Science, University of Macau, Macau SAR, China; 4 Tourism School, Hainan University, 58 Renmin Road, Haikou 570228, China

**Keywords:** tourism arrivals, tourism forecasting, fuzzy entropy, empirical wavelet transform, broad learning

## Abstract

This study proposes a decomposed broad learning model to improve the forecasting accuracy for tourism arrivals on Hainan Island in China. With decomposed broad learning, we predicted monthly tourist arrivals from 12 countries to Hainan Island. We compared the actual tourist arrivals to Hainan from the US with the predicted tourist arrivals using three models (FEWT-BL: fuzzy entropy empirical wavelet transform-based broad learning; BL: broad Learning; BPNN: back propagation neural network). The results indicated that US foreigners had the most arrivals in 12 countries, and FEWT-BL had the best performance in forecasting tourism arrivals. In conclusion, we establish a unique model for accurate tourism forecasting that can facilitate decision-making in tourism management, especially at turning points in time.

## 1. Introduction

Hainan Island is connected with the “Pan-Pearl River Delta”, Hong Kong, Macao, and Taiwan in the north, southeast Asian countries to the south, and Vietnam to the west (The People’s Government of Hainan Province [TPGoHP], 2012). Hainan Island was approved to set up the China (Hainan) Pilot Free Trade Zone by the Chinese government in 2018 which covers the whole island of Hainan. The overall plan of Hainan Province requires that the development of tourism, modern service industry, and high-tech industries take the lead, and the industrial layout of Hainan Island should be scientifically arranged. Therefore, it is very important to develop tourism on Hainan Island. Additionally, Hainan Island desires the establishment of an international tourism consumption center that can become an important engine of global economic growth. The international tourism consumption center shows much consumption, including a consumption environment and a world-class tourist attractions tourism complex, with a distribution center for both tourists in the locality and those abroad [1]. Artificial intelligence is widely employed in the development of high-quality tourism products and the growth of the tourism industry [2]. Accurate forecasting of tourist demand is imperative for academia and the tourism industries. In particular, accurate tourism arrival forecasting in Hainan Island can guide the administrative department in formulating policy. How to improve the forecasting performance when building the Hainan international tourism consumption center efficiently remains a challenge. Many studies [3,4] have declared that online search engine data could improve the tourism demands of forecasting performance. Zhang et al. [5] used an approach involving decomposition combined with prediction to experiment on a sample of tourists, mainly from Hong Kong. The modified method tapped into the good performance of the variational model decomposition in visitor prediction. Li et al. [6] developed a deep learning (DL) model with temporal feature learning capabilities for tourism volume data prediction by combining dimensionality reduction techniques; this produced a performance better than that of methods against which it was compared. Existing studies [4,7,8,9] have primarily established numerous techniques to improve the forecasting accuracy for tourism demand. Accuracy in tourism forecasting is critical for enabling administrative management to make appropriate decisions. In recent decades, decomposition ideas [10] have achieved better performance in the field of time series prediction. The signal decomposition methods represented by EWT [11] can fully exploit the submodular variables in the signal, thus providing a better pre-processing method for deep learning as a predictor for prediction. However, DL models require a large number of parameters and deep network structures, which invariably increase the computational complexity of the models [12]. Therefore, a new research idea has been to try to develop models that can achieve comparability with DL models without deep network structures [13,14]. 

At present, the development of the tourism business in Hainan, whether considered in proportion or considered in total, is relatively poor. Hainan urgently needs to break into the tourism business and build an international hub for tourism with high standards. Thus, we investigated a methodological approach by fusing decomposition and low-complexity DL networks and the broad learning (BL) system [15] for Hainan Island tourist forecasting. We combined artificial intelligence (AI) and tourism arrivals data for Hainan Island to analyze the Hainan Island arrivals of different foreign countries and provide more accurate forecasting for tourism.

The purpose of this study was to forecast the tourism arrivals of different foreign countries with an artificial intelligence (AI) model. The main aim was to compare and analyze the feasibility and effectiveness of this model developed based on decomposition and a non-deep deep learning (BL) framework. Experiments were conducted with a fusion-entropy EWT approach with the advantage of a typical neural network approach (BPNN) and BL approach for comparison. The main conclusion was that US foreigners had the most arrivals in 12 countries, and the FEWT-BL model performed the best in forecasting the tourist arrivals to Hainan from 12 countries. This should be helpful in identifying future directions of research on tourism arrival forecasting. Hence, this study provides advanced insights for researchers conducting future studies using AI models to forecast tourism demand.

## 2. Methodology

A total of 2592 observation data on Hainan Island arrivals were collected between January 2002 and December 2019 from the government’s official website. We compared the actual tourist arrivals from the US to Hainan with the predicted tourist arrivals using three models (FEWT-BL: fuzzy entropy empirical wavelet transform-based broad learning; BL: broad learning; BPNN: back propagation neural network). Broad learning forecasting models were implemented to predict the tourist arrivals to Hainan. The raw data were normalized and screened before analysis. Then, we applied an empirical wavelet transform (EWT) method to decompose the normalized tourist arrivals data. FEWT-BL is an improved performance method of EWT. This method aims to obtain R-square (R^2^), the root means square error (RMSE), individual intrinsic mode functions (IMFs), and mean absolute percentage error (MAPE). FEWT-BL was developed through the following steps.

### 2.1. Empirical Wavelet Transform

Jerome Gilles [11] first introduced EWT, which is defined as a set of bandpass filters that are selected through the spectral characteristics signal. To determine the frequency ranges of the bandpass filters, the Fourier spectrum signal is segmented. From the literature [16], a finite number of intrinsic modes for a time series can be effectively identified and extracted by the EWT. The EWT depends on robust pre-processing for peak detection and shows spectrum segmentation and establishes a related wavelet filter bank. The EWT algorithm steps include the signal extending, the Fourier transform executing, the boundaries extracting, the filter bank building and the sub-bands extracting.

The EWT computation can be shown as following [17]:

(1) The Fourier spectrum of the original precipitation series is segmented into N continuous segments. The limits are defined as $\omega_{n}$, where $\omega_{0}=0$ and $\omega_{n}=0$, respectively. Each segment is defined as $\Lambda_{n}=\left[\omega_{n-1},\omega_{n}\right]$. For each $\omega_{n}$, a transition phase $T_{n}$ with the width $2\tau_{n}$ is utilized. The range γ can be shown as:

(1) $$\gamma <\min_{n}\left(\frac{\omega_{n+1}-\omega_{n}}{\omega_{n+1}+\omega_{n}}\right)$$

(2) A series of empirical wavelets based on the Littlewood–Paley and Meyer’s wavelets is established. For $\forall_{n}>0$, the empirical scaling function and empirical wavelets can be shown by Equations (2) and (3), respectively:

(2) $${\overset{\wedge }{\phi }}_{n}\left(\omega \right)=\left\{\begin{array}{ll} 1 & \left|\omega \right|<\left(1-\gamma \right)\omega_{n}\\ \cos\left[\frac{\pi }{2}\beta \left(\frac{1}{2\tau n}\left|\omega \right|-\omega_{n}+\tau_{n}\right)\right] & \left(1-\gamma \right)\omega_{n}⩽\left|\omega \right|⩽\left(1+\gamma \right)\omega_{n}\\ 0 & \mathrm{otherwise} \end{array}\right.$$

(3) $${\overset{\wedge }{\varphi }}_{n}\left(\omega \right)=\left\{\begin{array}{ll} 1 & \omega_{n}\left(1+\gamma \right)⩽\left|\omega \right|⩽\left(1-\gamma \right)\omega_{n+1}\\ \cos\left[\frac{\pi }{2}\beta \left(\frac{1}{2\tau_{n+1}}\left|\omega \right|-\omega_{n+1}+\tau_{n+1}\right)\right] & \left(1-\gamma \right)\omega_{n+1}⩽\left|\omega \right|⩽\left(1+\gamma \right)\omega_{n+1}\\ \sin\left[\frac{\pi }{2}\beta \left(\frac{1}{2\tau }\left|\omega \right|-\omega_{n}+\tau_{n}\right)\right] & \left(1-\gamma \right)\omega_{n}⩽\left|\omega \right|⩽\left(1+\gamma \right)\omega_{n}\\ 0 & \mathrm{otherwise} \end{array}\right.$$

The function β(*x*) is defined as:

(4) $$\beta \left(x\right)=x^{4}\left(35-84x+70x^{2}-20x^{3}\right)$$

The inner products with the empirical scaling function achieved the approximation coefficients $W_{f}^{\varepsilon }\left(0,t\right)$ as follows:

(5) $$W_{f}^{\varepsilon }\left(0,t\right)=\langle f,\phi_{1}\rangle =\int f\left(\tau \right)\bar{\phi_{1}\left(\tau -t\right)}d\tau$$

The inner products with the empirical wavelets achieved the detailed coefficients $W_{f}^{\varepsilon }\left(n,t\right)$ as follows:

(6) $$W_{f}^{\varepsilon }\left(n,t\right)=\langle f,\psi_{n}\rangle =\int f\left(\tau \right)\bar{\psi_{n}\left(\tau -t\right)}d\tau$$

(3) The reconstruction series and empirical modes are shown as follows:

(7) $$f\left(t\right)=W_{f}^{\varepsilon }{\left(0,t\right)}^{*}\phi_{1}\left(t\right)+\sum_{n=1}^{N}W_{f}^{\varepsilon }{\left(n,t\right)}^{*}\psi_{n}\left(t\right)$$

(8) $$x_{0}\left(t\right)=W_{f}^{\varepsilon }{\left(k,t\right)}^{*}\phi_{1}\left(t\right)$$

(9) $$x_{k}\left(t\right)=W_{f}^{\varepsilon }{\left(k,t\right)}^{*}\phi_{k}\left(t\right)$$

### 2.2. Fuzzy Entropy

Entropy is a parameter in statistical thermodynamics that measures the degree of chaos in a system to represent the state of matter [18,19]. The concepts of information entropy, sample entropy, and approximate entropy have been successively proposed. Fuzzy entropy is much more efficient and it can improve the sample entropy algorithm that is proposed in the literature [20]. The fuzzy entropy algorithm selects the exponential function as the fuzzy function to measure the similarity between two variables and has superior signal measurement properties compared to approximate entropy and sample entropy. They include relative consistency, noise resistance, and better continuity [21]. Therefore, this study combined fuzzy entropy with the EWT technique to extract signal features, and then construct feature vectors. First, the sequences are defined as follows:

$$X\left(i\right)=\left[x\left(i\right),x\left(i+1\right),\ldots x\left(i+m-1\right)\right]-x_{0}\left(i\right),\;x\left(i\right)=\frac{1}{m}\sum_{k=0}^{m-1}x\left(i+k\right)$$

where $x_{0}\left(i\right)$ stands for *m* consecutive $x\left(i\right)$.

$d_{ij}^{m}$ is defined as the distance between $X\left(i\right)$ and $X\left(j\right)$, and $d_{ij}^{m}$ is the maximum absolute value of the difference between the two corresponding elements; that is:

$$d_{\mathrm{ij}}^{m}=\underset{k\in \left(0,m-1\right)}{\max}\left\{\left|u\left(i+k\right)-u_{0}\left(i\right)-(u\left(j+k\right)-u_{0}\left(j\right)\right|\right\}$$

where I, j = 1, 2, …, N − m, i ≠ j.

The fuzzy similarity is defined by the fuzzy function; that is:

$$D_{\mathrm{ij}}^{m}=e^{-{\left(d_{\mathrm{ij}}^{m}/r\right)}^{n}}$$

where n and r represent the gradient and width of the boundary, respectively.

Similarly, the function ϕ is defined as:

$$\varphi_{\left(n,r\right)}^{m}=\frac{1}{N-m}\sum_{i=1}^{N-m}(\frac{1}{N-m-1}\sum_{j=1,j\neq i}^{N-m}D_{\mathrm{ij}}^{m})$$

Then, 1 is added to the dimension and is turned into m+1; the above steps are repeated to obtain $\varphi_{\left(n,r\right)}^{m+1}$.

The fuzzy entropy (Fuzzy En) of the signal sequence is:

$$\mathrm{FuzzyEn}\left(m,n,r\right)={\ln\varphi }_{\left(n,r\right)}^{m}-\varphi_{\left(n,r\right)}^{m+1}$$

In fuzzy entropy, r represents the width of the fuzzy function boundary; too large an r will result in the loss of much statistical information, and too small an *r* results in a failure to estimate the statistical properties as well and increases sensitivity to the resulting noise. Normally, r is taken to be between 0.1 and 0.25 *SD*(*x*) (where *SD*(*x*) is the standard deviation of the series). For the choice of n, which determines the gradient of the similarity tolerance bound, the larger the n, the larger the gradient. n plays a weighting role in the calculation of similarity between fuzzy entropy vectors. To capture as much detailed information as possible, one is generally advised to use smaller integer values. Thus, this study selected m = 2, r = 0.15 × *SD*, and n = 2.

### 2.3. Broad Learning System

The broad learning system (BLS) broadly extends the network [22]. Considering the general supervised learning task, the training data set is given as $\left\{(X,\overset{\wedge }{Y})|X\in {\mathbb{R}}^{N\times D},Y\in {\mathbb{R}}^{N\times C}\right\}$ from *C* classes, where each row in *X* and *Y* denotes the data point *x_i_* = (*x_i_*_1,_ *x_i_*_2, …,_ *x_iD_*) and target vector *y_i_* = (*y_i_*_1,_ *y_i_*_2, …,_ *y_iC_*), respectively [23]. In BLS, a random mapping generation with *n* nodes can be defined as follows:

(10) $$Z_{i}=f_{i}(XW_{ei}+\beta_{ei}),i=1,2,\ldots ,N_{w}$$

where the weights *W_et_* and the bias term $\beta_{ei}$ are randomly determined with the proper dimensions. The whole feature nodes can be defined as $Z^{n}\equiv [Z_{1},\ldots ,Z_{n}]$, and the *m^th^* group of enhancement nodes can be computed as:

(11) $$Z^{n}\equiv \xi (Z^{n}W_{hm}+b_{hm})$$

where ξ is a nonlinear activation function, and the outputs of the enhancement layer can be denoted by $H^{m}≜[H_{1},H_{2},\ldots ,H_{m}]$.

Overall, the formula of the broad learning model can be deduced as follows:

(12) $$\overset{\wedge }{Y}=[Z_{1},Z_{2},\ldots ,Z_{n}|H_{1},H_{2},\ldots ,H_{m}]W=[Z^{n}|H^{m}]W=AW$$

where $A=[Z^{n},H^{m}]$, and *W* is the output weight connection of feature nodes and enhancement nodes to the output layer. *W* could be obtained by minimizing the objective:

(13) $$\arg\underset{W}{\min}\Gamma_{\mathrm{BLS}}=||Y-AW{\left|\right|}^{2}+\lambda \left|\right|W{\left|\right|}^{2}$$

where the first term denotes the training errors, and the second term is a regularization term and λ is a regularization parameter to balance the influence of error terms and the model complexity. According to a simple derivative operation on W, we can obtain:

(14) $$W={\left(A^{T}A+\lambda I\right)}^{-1}A^{T}Y$$

The BLS output weight W is always obtained as the matrix $\left(A^{T}A+\lambda I\right)$.

This work combined the role of fuzzy entropy in signal decomposition. First, the corresponding components were obtained by the EWT decomposition of tourism data. Then, the energy value of each component was calculated using fuzzy entropy, and the information entropy of each component was calculated by taking the percentage of each component in the total amount as the probability density function to obtain updated estimates. Finally, the BL model was used for prediction.

## 3. Results and Discussion

### 3.1. Model Performance Evaluation

Two error measure indexes were utilized in the forecasting experiments to assess the prediction performance among the involved models. The indexes were the mean absolute percentage error (MAPE), and the root means square error (RMSE).

The indexes were shown as:

(15) $$\mathrm{MAPE}=\frac{1}{N}\sum_{i=1}^{N}\frac{\left|q_{p}(i)-q_{o}(i)\right|}{q_{o}(i)}\times 100\%$$

(16) $$\mathrm{RMSE}=\sqrt{\frac{1}{N}\sum_{i=1}^{N}{\left(q_{p}(i)-q_{o}(i)\right)}^{2}}$$

### 3.2. Results of Decomposition

**Figure 1:**
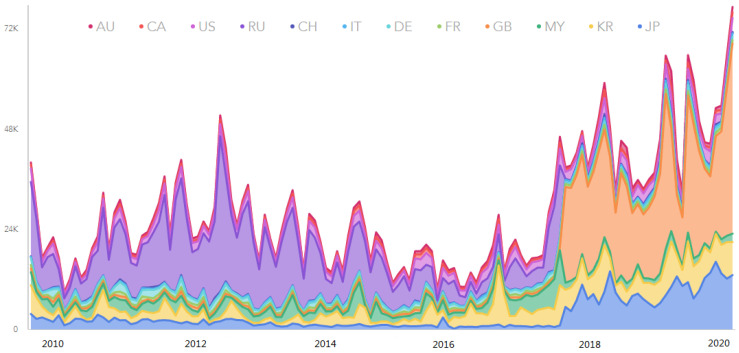
The number of foreign tourists received by tourist hotels in Hainan province (by country/region). Note: AU: Australia, US: United States of America, CA: Canada, RU: Russia, CH: Switzerland, IT: Italy, DE: Germany, FR: France, GB: Great Britain, MY: Malaysia, KR: Korea, JP: Japan.

We collected data on tourist arrivals to Hainan province from 12 countries, as shown in the legend of Figure 1 to study the predictive ability and improved the accuracy of tourism arrivals forecasting. Figure 1 shows the number of tourists from different countries/regions traveling to Hainan province by month, where the number of US foreign arrivals is the highest. All data information was collected from the Hainan Province Tourism Board. The abbreviations AU, US, CA, RU, CH, IT, DE, FR, GB, MY, KR, and JP are used to represent the 12 counties.

**Figure 2:**
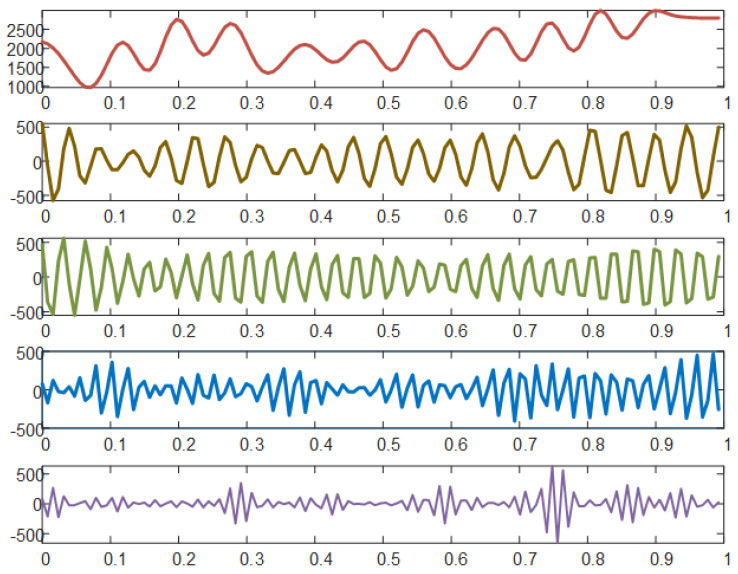
Components of US tourists received by tourist hotels in Hainan province. The components were decomposed and preprocessed by EWT. The five components from top to bottom are IMF 1 to IMF 5. IMF, intrinsic mode function; EWT, empirical wavelet transforms.

We applied the empirical wavelet transforms (EWT) to decompose the original data set into individual components. The intrinsic mode function (IMF) is the modulated function which is amplitude-frequency. Five IMFs and one residual item were analyzed by MATLAB. The results declared various representations of each tourist arrivals component, as shown in Figure 2. First, from the perspective of component semantic interpretation, the main difference between these components is the frequency of occurrence. All the components of the tourist arrivals data set present distinct frequencies. IMF 5 has the highest frequency, whereas IMF 1 has the lowest. The more frequent the IMF, inevitably, the greater the amounts of information and noise. The individual tourist arrivals components show the cycles, trends, and seasonal patterns. Similarly, IMF 1 and IMF 2 could be thought of as secondary cycles of data, during which there are other distinct peaks and valleys. IMF 4 is thought of as modest fluctuations, which contain less information. On the contrary, the residual is of certainty long-term behavior, which can suggest the trend of the tourism market in the long-term. Huang et al. [24] also believed that the residual component could determine long-term behavior. 

**Figure 3:**
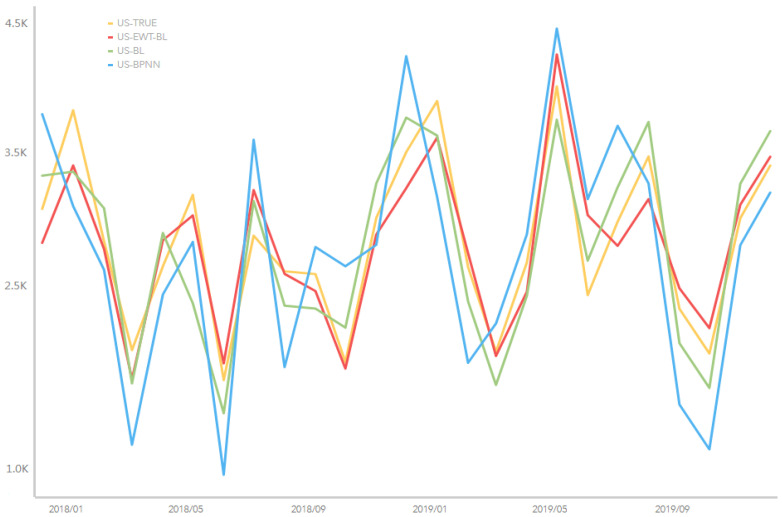
The predictive performance of four models for the actual tourist arrivals to Hainan from the US country.

As indicated in Figure 3, we used the average period formula to calculate the average period IMF 3 displayed in all the tourist arrivals data, which was about 12 months. Therefore, IMF 3 was thought of as the main tourist arrivals cycle, which declared the main valleys and peaks.

### 3.3. Analysis of Forecasting Results

After the decomposition of the Hainan province tourist arrivals data, the decomposed components were predicted, respectively, and this comprehensive prediction was then combined to achieve the result. In our experiment, all data sets were grouped into the training set and prediction set, with the first 13 years of data from 18 years as the training set and the last 5 years of data as the prediction set. Additionally, a rolling method was used, and the step size was set to 1.

**Figure 4:**
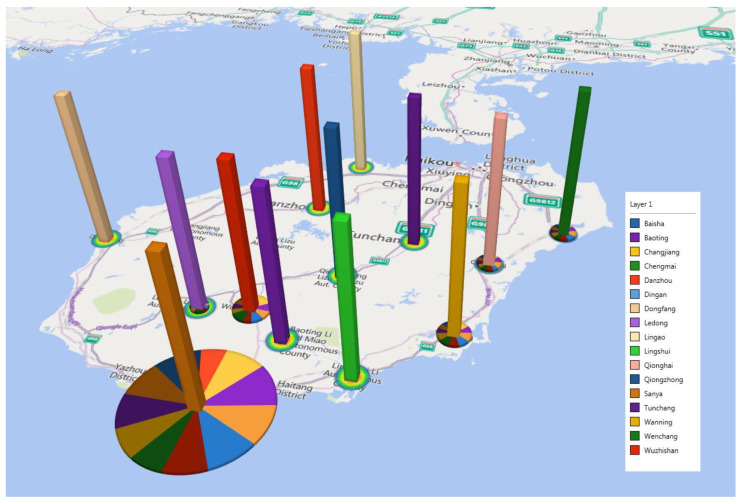
The reception of inbound tourists by cities and counties in Hainan province. Note: The area of the circle represents the reception of inbound tourists; different color pieces in the circle represent the various countries of foreigners.

For example, Figure 4 depicts the reception of inbound tourists by cities and counties in Hainan province; Sanya city has the optimal reception condition with foreigners from various countries. Therefore, the optimal reception condition of Sanya city can stimulate its tourism economy and promote its international development. Figure 4 shows the predictive ability of the FEWT-BL method compared to the non-decomposed method and back propagation neural network (BPNN) for tourist arrivals to Hainan, using tourists from the US as an example. We can see from Figure 4 that the yellow line indicates the true data, and the red, green, and blue lines represent the results of the three compared methods. It is easy to identify that the red line (FEWT-BL) is closer to the yellow line (TRUE) than the green (BL) and the blue line (BPNN), which means that the proposed FEWT-BL method obtained the best performance compared to the other methods. These peak turning points show the direct change in Hainan Island tourist arrivals. For example, from May 2018 to September 2018 and from September 2018 to January 2019, Hainan Island tourist arrivals from the United States (US) showed a downturn and uptrend, including the valley and peak point. From May 2019 to September 2019, tourist arrivals from the US experienced a downtrend, including the turning point. Therefore, Hainan administrators should pay attention to the fluctuations in the tourism market and make prompt decisions to ensure stable and upward growth in the tourism industry. Based on this forecasting result, our study can support decision-making for policy administration in tourism, especially at turning points in time.

More detailed results with all the predicted performances for 12 countries are shown in Table 1. Evaluation metrics (RMSE and MAPE) were utilized to assess the performance of the benchmark methods. Compared with the BL and BPNN methods, the proposed method—the EWT-based BL algorithm—almost obtained the lowest RMSE and MAPE. The R^2^ is depicted the agreement extent between forecasting and training tourist data. The FEWT-BL method is much more preferable than the BL without EWT technology in terms of R^2^. The results indicate that the proposed FEWT-BL method was favorable for predicting tourism arrivals compared with other BL and BPNN methods. As indicated in Table 1, the FEWT-BL forecasting accuracy for the tourist arrivals from Italy was higher (R^2^ = 0.96) than that of the BL (R^2^ = 0.93) and BPNN (R^2^ = 0.88) models. Alternatively, the FEWT-BL forecasting accuracy for the tourist arrivals from the United States performed better (R^2^ = 0.94) compared to the BL (R^2^ = 0.91) and BPNN (R^2^ = 0.89) models. The DM test was used to examine whether there was a significant difference between the predictive accuracy of the two models. The results of the DM test in Table 2 showed that, among the three prediction models, FEWT-BL outperformed the remaining two. In summary, the results indicate that the FEWT-BL method can help to reduce forecasting errors with original official government data. Particularly, the FEWT-BL model can accurately achieve predictive ability for tourist arrivals.

**Figure 5:**
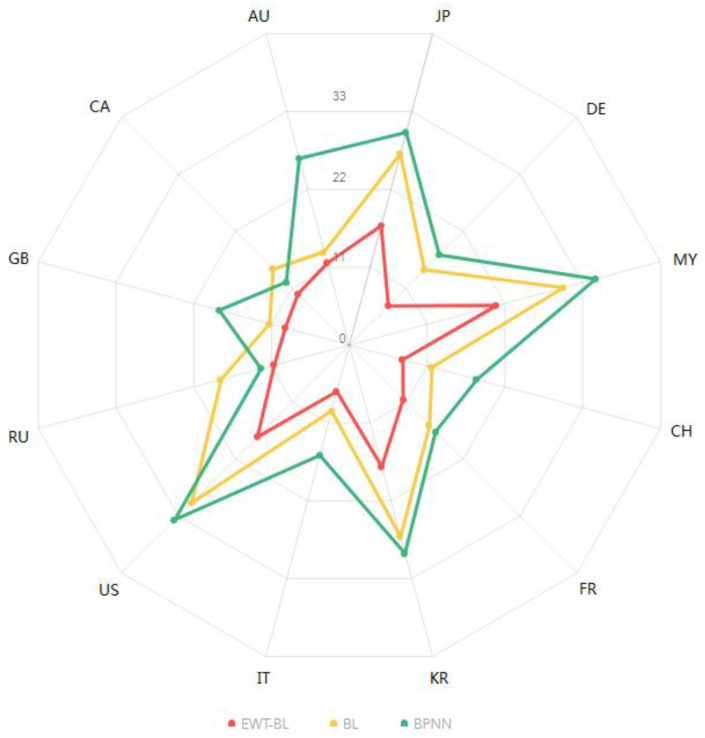
Radar chart of RMSE values for tourist arrivals to Hainan from 12 countries for different methods. Note: RMSE: root mean square error, FEWT-BL: empirical wavelet transform-based broad learning, BL: broad learning, BPNN: back propagation neural network.

To present the results more visually, we used radar diagrams to show the performance of the three methods, where the red, yellow, and green lines in Figure 5 represent the FEWT-BL, BL, and BPNN methods, respectively. The red line is completely contained in the innermost layer, which means that the FEWT-BL method, represented by the red line, achieves the best RMSE performance. The yellow line is mostly wrapped up in the green line, and only a small number, two points, intersect with the green line, meaning that the BL method called the BPNN model performed better in most cases. Many researchers [25,26,27,28] have shown that the tourism perspective is emphasized ethnic minorities Additionally, Han et al. [29] studied Halal tourism, including travel motivation and customer desire. Many researchers are seeking new technologies to manage tourism [30]. Our study indicates that US foreigners had the most arrivals in 12 countries, and the FEWT-BL performed the best in forecasting the tourist arrivals to Hainan from 12 countries. The results show that the FEWT-BL method has a much preferable predictive ability in forecasting turning points. Therefore, we developed an accurate FEWT-BL method to forecast tourist arrivals to Hainan from 12 countries. We recommend using this method in the future for forecasting tourism to achieve improved performance.

## 4. Conclusions

Two strengths of this study are worth highlighting: Firstly, the updated broad learning (FEWT-BL) approach can accurately forecast tourism arrivals and reception, which can facilitate decision-making in tourism management, especially at turning points in time. Secondly, this study illustrates how a proposed decomposed broad learning model can improve the forecasting accuracy for tourism arrivals on Hainan Island, which has rarely been used for tourist arrivals. Hence, this study provides advanced insights for researchers seeking to conduct future studies using AI models to forecast tourism demand. Additionally, this method of forecasting through AI could be strongly recommended for applications in tourism. In this work, we focused on the prediction performance for tourism data based on machine learning and lightweight deep neural networks. The comparisons included a comparison between the decomposed and the traditional shallow neural network, as well as a comparison with the non-decomposed model. Therefore, this manuscript highlights the role before and after the decomposition and the forecasting ability of the new network structure compared to the traditional network structure. In future work, we will continue to compare and analyze the model proposed in this paper and some classical time series prediction methods.

## Figures and Tables

**Table 1:** Forecasting performance of the models for tourist arrivals to Hainan from 12 countries.

Country	FEWT-BL			BL			BPNN		
	RMSE	MAPE	R^2^	RMSE	MAPE	R^2^	RMSE	MAPE	R^2^
JP	16.86	4.45	0.93	27.04	6.44	0.86	30.06	12.58	0.84
KR	7.56	2.68	0.95	14.54	3.67	0.92	17.41	4.46	0.9
MY	20.87	11.05	0.91	30.3	12.68	0.87	34.88	14.24	0.81
GB	7.6	10.96	0.95	11.7	12.02	0.92	18.07	14.24	0.89
FR	10.42	1.4	0.94	15.54	1.37	0.89	16.7	1.99	0.88
DE	17.18	1.14	0.92	27.07	1.23	0.86	29.44	1.48	0.85
IT	6.54	0.71	0.96	9.3	1.41	0.93	15.57	1.6	0.88
CH	17.75	0.62	0.92	30.43	1.8	0.85	33.69	2.33	0.82
RU	10.6	1.16	0.93	18.1	2.27	0.9	12.48	2.11	0.91
US	9.04	0.31	0.94	11.26	1.3	0.91	18.38	2.19	0.89
CA	9.89	0.83	0.94	14.66	1.77	0.92	12.16	1.45	0.91
AU	11.57	1.12	0.92	13.18	1.33	0.91	26.37	2.66	0.87

Note: RMSE, root mean square error; MAPE, mean absolute percentage error; R^2^, R-square

**Table 2:** DM test results for tourist arrivals to Hainan from the US.

Models	BPNN and BL	FEWT-BL and BL	FEWT-BL and BPNN
DM value	1.4209	5.9589	4.0377
*p*	0.1553	2.5394 × 10^−9^	5.3978 × 10^−5^

## Data Availability

All data were present in the study.
